# Design considerations for developing measures of policy implementation in quantitative evaluations of public health policy

**DOI:** 10.3389/frhs.2024.1322702

**Published:** 2024-07-15

**Authors:** Natalie Riva Smith, Douglas E. Levy, Jennifer Falbe, Jonathan Purtle, Jamie F. Chriqui

**Affiliations:** ^1^ Department of Social and Behavioral Sciences, Harvard TH Chan School of Public Health, Boston, MA, United States; ^2^ Mongan Institute Health Policy Research Center, Massachusetts General Hospital, Boston, MA, United States; ^3^ Harvard Medical School, Boston, MA, United States; ^4^ Human Development and Family Studies Program, Department of Human Ecology, University of California, Davis, CA, United States; ^5^ Department of Public Health Policy & Management, Global Center for Implementation Science, New York University School of Global Public Health, New York, NY, United States; ^6^ Institute for Health Research and Policy, University of Illinois Chicago, Chicago, IL, United States; ^7^ Department of Health Policy and Administration, School of Public Health, University of Illinois Chicago, Chicago, IL, United States

**Keywords:** health policy, implementation science, policy research, policy implementation science, policy evaluation

## Abstract

Typical quantitative evaluations of public policies treat policies as a binary condition, without further attention to how policies are implemented. However, policy implementation plays an important role in how the policy impacts behavioral and health outcomes. The field of policy-focused implementation science is beginning to consider how policy implementation may be conceptualized in quantitative analyses (e.g., as a mediator or moderator), but less work has considered how to measure policy implementation for inclusion in quantitative work. To help address this gap, we discuss four design considerations for researchers interested in developing or identifying measures of policy implementation using three independent NIH-funded research projects studying e-cigarette, food, and mental health policies. Mini case studies of these considerations were developed via group discussions; we used the implementation research logic model to structure our discussions. Design considerations include (1) clearly specifying the implementation logic of the policy under study, (2) developing an interdisciplinary team consisting of policy practitioners and researchers with expertise in quantitative methods, public policy and law, implementation science, and subject matter knowledge, (3) using mixed methods to identify, measure, and analyze relevant policy implementation determinants and processes, and (4) building flexibility into project timelines to manage delays and challenges due to the real-world nature of policy. By applying these considerations in their own work, researchers can better identify or develop measures of policy implementation that fit their needs. The experiences of the three projects highlighted in this paper reinforce the need for high-quality and transferrable measures of policy implementation, an area where collaboration between implementation scientists and policy experts could be particularly fruitful. These measurement practices provide a foundation for the field to build on as attention to incorporating measures of policy implementation into quantitative evaluations grows and will help ensure that researchers are developing a more complete understanding of how policies impact health outcomes.

## 1 Introduction

Public policy plays a major role in improving public health, and most of the greatest public health achievements have resulted from policy action (1, 2). Disciplines such as economics, public administration, political science, and health services research have a long history of evaluating policies and quantifying their effects on health and related outcomes. Methodologically, this is typically operationalized by comparing outcomes in jurisdictions with and without a policy, treating the presence/absence of policies as binary conditions, and using difference-in-differences analyses or related quasi-experimental causal inference methods (3).

However, this approach masks potentially important variability in components of policy implementation, which in turn can affect outcomes and researchers' ability to draw clear conclusions about a policy's effects (3–6). For example, similar policies can have different provisions that affect implementation. One state with retail restrictions on electronic cigarettes may empower state public health officials to enforce the restrictions, while others may rely on local public health or law enforcement officials to ensure compliance. Likewise, variation in funding for policy enforcement and government capacity for monitoring compliance can result in heterogeneity in implementation and subsequent outcomes. In addition, even when jurisdictions (e.g., states) have the exact same provisions in their policy, how the policy is interpreted and implemented may vary based on the entity responsible for implementation. In one published example, McGinty et al*.* found that state opioid prescribing laws did not significantly change opioid prescriptions or nonopioid pain treatments (7). Their team's parallel qualitative work describing suboptimal implementation and limited penalties for nonadherence among these laws helped to put the quantitative findings into greater context and provided explanation as to why their findings did not change clinical practice (7, 8).

These qualitative studies demonstrate the importance of conducting research to understand policy implementation, but no measures of policy implementation were included in the quantitative components. Inclusion of quantitative measures of policy implementation, combined with qualitative findings, can generate a more holistic understanding of the mechanisms or processes that contribute to policy impact (3, 6, 9). Recent work has discussed that policy implementation can be conceptualized as an effect modifier or a mediator (6); such analyses are promising analytic approaches that are compatible with standard regression modeling methods.

Using such analytic approaches ultimately depends on being able to measure policy implementation in a rigorous way, and there is a major lack of valid and reliable measures of health policy implementation determinants, processes, and outcomes (10–15). As a starting point to address the policy implementation measurement gap, provide guidance for researchers designing studies, and ultimately improve how policy implementation is analytically incorporated into quantitative policy evaluation research, we discuss four considerations for developing or identifying existing measures of policy implementation.

## 2 Methods

We identified our considerations by drawing on three currently funded NIH research grants that focus on policy implementation across areas of health at the state or local levels, as well as our team's expertise and subject matter knowledge. While these three grants all focus on studying policy implementation and include a quantitative component, their designs are diverse and offer potentially informative comparisons. A key thread linking all of these studies is that all focus on better understanding the “black box” of implementation after a policy has been adopted (16).

Our team met periodically to discuss the development of our considerations and design decisions. Each project lead first completed an implementation research logic model [IRLM, (17)] for their research grant. Group meetings were structured around discussing different components of the IRLM (e.g., determinants, implementation strategies, or implementation outcomes). We additionally used existing implementation science theories, models, or frameworks to guide these discussions. Specifically, we relied on the Consolidated Framework for Implementation Research (18, 19), the Expert Recommendations for Implementing Change compilation of implementation strategies (20), the Bullock et al*.* policy determinants and process model (21), and the Proctor Implementation Outcomes Framework (22, 23). These helped guide our group discussions and allowed the team to extract key information related to each study in a consistent format. Through group discussions, we iteratively developed a list of key considerations for developing or identifying measures based on commonalities across the three funded studies as well as our combined expertise in quantitative policy evaluation, public policy, and dissemination and implementation science.

### 2.1 Descriptions of included studies

The VAping POlicy Research (VAPOR) study seeks to (1) characterize the implementation of e-cigarette policies, (2) estimate the impact of these policies – accounting for strength of implementation – on e-cigarette, combustible cigarette, and cannabis use, and (3) project the future impact of alternate policy configurations using simulation modeling.

The Berkeley Choices and Health Environments at ChecKOUT (CHECKOUT) study focuses on the world's first healthy checkout policy, which prohibits the placement of high-added-sugar and high-sodium products and encourages healthy foods and beverages at store checkout areas. This work aims to (1) assess how the policy impacts store food environments, (2) how the policy impacts food purchasing, and (3) examine implementation factors that influence the effectiveness of the policy.

The 988 Lifeline financing study is focused on how states are supporting the implementation of a new three-digit dialing code for the national 988 Suicide & Crisis Lifeline, which was created by a federal law. The study aims to (1) characterize how states are financing 988 implementation, (2) explore perceptions of the financing determinants of 988 implementation success and understand the acceptability and feasibility of different financing strategies, and (3) examine how financing strategies affect policy implementation, mental health crisis, and suicide outcomes.

## 3 Results

We show key information from each study's design and IRLM in Table 1 and each completed IRLM is included in the Supplementary Appendix. Through our discussions of the IRLMs, we identified four key considerations to developing and identifying measures that researchers may consider as they plan and execute quantitative policy implementation studies. For each consideration discussed below, we provide illustrative examples from each study and provide a summary of the considerations in Table 2.

**Table 1:** Key policy implementation study components.

Study component	VAPOR	CHECKOUT	988
Policy(ies)	Six state e-cigarette policies: minimum legal sales age, flavor restrictions, taxes, clean indoor air laws, sales restrictions, licensure requirements	Healthy checkout policy that sets nutrition standards for foods and beverages displayed in store checkout lanes	Federal law that created three-digit dialing code for crisis line, expanded scope of crisis line, and delegated implementation financing responsibility to states
Level	State policies, state implementation	Local policy, local implementation (Berkeley, California)	Federal policy, state implementation
Main focus of study	Identifying implementation strategies for state e-cigarette policies and incorporating strength of policy implementation into quantitative models	Assessing impact of a local policy on food environments and consumer behaviors and understanding the policy implementation process	Determining effects of state implementation financing strategies and understanding policy implementation processes
Key implementation outcomes	Tax revenue collected, enforcement actions taken, inspection and sting operations, total budget for implementation (not measured in this study)	Degree of retailer compliance with healthy checkout policy	988 call volume and answering rates
Key service outcomes	n/a	Healthfulness of food environments	Timely receipt of quality crisis and mental health services
Key effectiveness outcomes (behavior and health)	Youth and adult e-cigarette use, youth and adult use of combustible tobacco products (e.g., cigarettes) and cannabis	Consumer purchases and dietary intake	Suicide attempts, suicide deaths, and emergency department visits for mental health crises and self harm
Data sources/collection methods	Qualitative interviews with state agencies, short implementation survey, CDC and state Behavioral Risk Factor Surveillance System data	Qualitative interviews and short survey with city staff and officials, community organizations, and store owners and managers; primary data collection of retailer compliance	Qualitative interviews with policy implementers, call volume and routing data from Vibrant Emotional Health, CDC mortality data, National Survey on Drug Use and Health, Agency for Healthcare Research and Quality's State Emergency Department database
Primary analytic methods	Thematic analysis of qualitative interviews; Difference-in-differences modeling; Microsimulation modeling	Descriptive analysis of qualitative interviews; Difference-in-differences and synthetic control modeling	Descriptive analysis of qualitative interviews and quantitative survey data; Difference-in-differences analyses
Practice partners	Public Health Law Center, the Truth Initiative, and the Tobacco Control Network (TCN) of the Association of State and Territorial Health Officials	Public health advocate and community leader who helped design the policy	Vibrant Emotional Health, which coordinates the 988 Lifeline, American Foundation for Suicide Prevention, Association of State and Territorial Health Officials

**Table 2:** Design considerations for measuring policy implementation.

Consideration	Rationale
1. Clearly specify the implementation logic of the policy under study	Clear implementation logic is required to fully understand the policy being studied (e.g., conceptualization, mechanisms) and develop or identify appropriate measures.
2. Develop an interdisciplinary team consisting of policy practitioners and researchers with expertise in quantitative methods, public policy and law, implementation science, and subject matter knowledge.	The complexity of policy implementation measurement necessitates an interdisciplinary team to ensure that measures are theoretically and practically sound.
3. Use mixed methods to identify, measure, and analyze relevant policy implementation determinants and processes.	Mixed methods are critical to appropriate measurement development because they support a purposeful integration of policy implementation determinants and processes into quantitative analyses.
4. Build flexibility into project timelines to manage delays and challenges due to the real-world nature of policy.	Measuring policy implementation and including it in quantitative analyses will typically require researchers to be account for delays and challenges encountered during policy implementation.

### 3.1 Clearly specify the implementation logic of the policy under study

Differences between the three projects underscored that policy implementation cannot be measured with a one-size-fits-all approach. Each policy area is unique, with different policy actors, contexts, and goals - the who, what, how, and why - similar to recommendations for specifying implementation strategies (24). To appropriately identify or develop measures of policy implementation, it is important to clearly define the policy under study, its hypothesized mechanisms of action, and which implementation determinants and outcomes are relevant. Therefore, a key consideration we identified was for investigators to clearly specify the implementation logic of their policy, using existing tools and frameworks from implementation science (including the IRLM) and related fields (e.g., public administration research, political science). Having this implementation logic specified can help ensure that there is conceptual alignment between the policy exposure, implementation outcomes, and behavioral or health outcomes (25). Beyond conceptual alignment, clear specification can also help define the statistical role different elements of the study may play (e.g., mediator, moderator, confounder). Specifying these elements is essential to understand what variables are needed to sufficiently specify statistical models and identify what needs to be measured to statistically identify an effect of interest with a reasonable degree of precision. This in turn enables researchers to identify what type of measurement tools or approaches are needed, identify existing measures in the literature, and understand if new measures are needed. Measures can be derived from routinely collected administrative data or primary data collection.

#### 3.1.1 VAPOR

The VAPOR study is specifically interested in understanding the variety of implementation strategies for different e-cigarette policies and identifying simple ways to measure the strength of policy implementation. Studying the simultaneous implementation of more than one policy means that the measurement of policy implementation cannot be policy specific. For that reason, the team developed two short questions that will be fielded to the relevant individuals within state governments: (a) the degree to which policies are implemented as written, and (b) whether they have adequate resources to implement the policies. The team has also had to make choices about classifying policies for the purposes of analyses. There is much heterogeneity between policy details, separate from implementation heterogeneity (e.g., some states with flavor restrictions prohibit sales of all flavored e-cigarettes while others allow sales of mint or menthol products). In studies with large sample sizes, this could be managed by including a variety of analytic variables capturing such heterogeneity, but in evaluations of state policies, sample sizes are limited. Thus, the VAPOR team has had to wrestle with how to collapse similar policies across states into meaningful categories while maintaining sample sizes that are needed for analyses.

**Figure 1:**
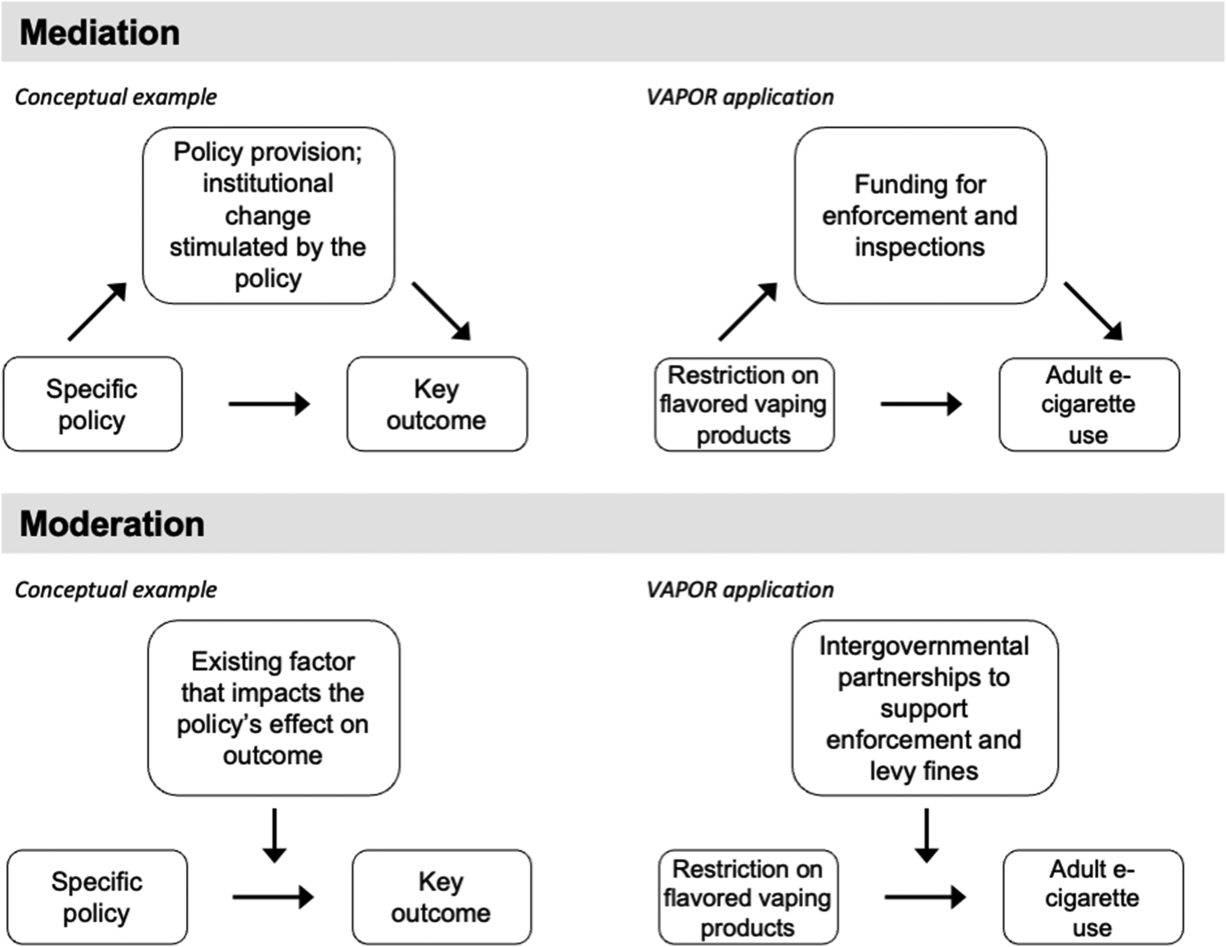
Example of policy implementation conceptualized as a mediator or moderator.

As an example of how clearly specifying implementation logic can assist with conceptual clarity and model specification, the VAPOR team used the IRLM process to interrogate what mechanisms (a specific component of the IRLM) would operate as mediators or moderators. Through this process, consensus emerged that moderators are typically contextual elements (inner/outer setting) that affect the relationship between the policy and outcomes, while mediators are typically factors that lie along the causal pathway between the policy and outcomes (generally institutional changes that are directly caused by the policy and its implementation strategy). Specificity is crucial to determine what is conceptually a mediator vs. a moderator (6). We provide a general illustration of this conceptualization as well as a specific example from VAPOR in Figure 1.

#### 3.1.2 CHECKOUT

In comparison to VAPOR, the CHECKOUT study focuses on the implementation of a single policy (a healthy checkout ordinance), implemented in a single jurisdiction (the city of Berkeley), and the first implementation study of this type of policy. As such, their study delves much deeper into the specifics of how the healthy checkout policy is being implemented. In-depth interviews with key stakeholders, combined with a brief quantitative survey, will generate rich data that can be used to develop and refine a broader set of quantitative measures to examine implementation heterogeneity across jurisdictions once healthy checkout policies are more widely adopted.

Although this is the first implementation study of a municipal healthy checkout policy, there are parallels between this policy and others (e.g., SSB excise taxes and restrictions on tobacco placement) that are helpful in developing an intuitive logic model. First, there is evidence from field experiments and voluntary policies that improving the healthfulness of checkouts also improves the healthfulness of consumer purchases (26). Second, prior evaluations of policy implementation have identified the importance of the following for improving health behaviors and outcomes: effective communications with retailers (e.g., definitions and lists of compliant and non-compliant products) (27), retailer compliance (e.g., the extent to which they stock only compliant products at checkout) (28), and enforcement and fines (29). Although this study is assessing implementation in a single city, the researchers expect to observe variability in how store managers and owners understand, interpret, and buy into the policy, and hence their store's compliance. Variability in compliance may also be observed over time based on the timing and robustness of the city's inspections, communications, and fines. The researchers' annual in-store assessments of products at checkout will provide objective quantitative measures of compliance, while the store interviews will indicate reasons for variability in compliance across stores and time. These data will not only inform constructs to assess in future quantitative measures of healthy retail policy implementations, but may also inform how to improve the next healthy checkout ordinance and its implementation.

#### 3.1.3 988 Lifeline

The 988 study focuses specifically on one state financing strategy to support the implementation of the 988 Suicide & Crisis Lifeline: 988 telecom fees. Telecom fees, which are adopted by state legislatures, were identified in the federal law that created 988 as the recommended financing strategy that states should use (though there is no requirement for them to use it). These telecom fees–which are flat monthly fees on cell phone bills (e.g., 30 cents a month)–are consistent with how the 911 system in the United States is in part financed. As of March 2024, eight states had adopted 988 telecom fees. The study conceptualizes the state laws that create the fees as implementation strategies to support federal policy implementation and increase the reach of services provided by the 988 Suicide & Crisis Lifeline. The policies are operationalized as a dichotomous variable (988 telecom fee passed in the state, yes/no) as well as a continuous variable (dollar amount of revenue the 988 telecom fee generated annually per state resident).

### 3.2 Develop an interdisciplinary team consisting of policy practitioners and researchers with expertise in quantitative methods, public policy and law, implementation science, and subject matter knowledge

The projects illustrate the importance of involving an interdisciplinary team when measuring policy implementation. All study teams included researchers with expertise in methods and models used to evaluate health policies (e.g., difference-in-differences analyses, epidemiological and econometric methods), legal and policy expertise, implementation science, and health subject matter expertise. Implementation science expertise is critical to the clear conceptualization of different components of policy implementation, including the distinction between determinants, implementation strategies, mechanisms, processes, and outcomes – something that the team found difficult throughout our group discussions for this paper. The importance of mixed methods to policy implementation measurement (see next section “Use mixed methods…”) also necessitates team expertise with qualitative, quantitative, and mixed methods approaches. Practice partners can help guide the recruitment of individuals who are best able to provide information on policy implementation components, and support other aspects of data collection or access. Including practice partners also ensures that there is a built-in feedback loop to communicate findings to other policy practitioners who may be considering or implementing similar policies.

#### 3.2.1 VAPOR

The VAPOR team is led by a health policy and health services researcher with expertise in tobacco control and implementation science. Additional investigators and consultants bring expertise in tobacco and e-cigarette policy, addiction medicine, youth vaping, qualitative methods, implementation science, statistics (difference-in-differences methods), and simulation modeling. The team's tobacco and e-cigarette control experts come not just from the study's research center, but from major national organizations, including the Public Health Law Center, the Truth Initiative, and the Tobacco Control Network (TCN) of the Association of State and Territorial Health Officials. These team members help guide the execution of the research, including the recruitment of key implementation actors in different states. Representatives from these organizations constitute an important advisory board that is also called on to help guide analyses - for example, using their practice-based knowledge to help identify what policy synergies are useful to probe for in analyses, both independent and dependent on implementation.

#### 3.2.2 CHECKOUT

The CHECKOUT study is led by a nutritional epidemiologist with experience evaluating food policies and their implementation processes using mixed methods. Other investigators include health economists (difference-in-differences and synthetic control methods) and behavioral scientists, and investigators are working with the director of a food policy NGO and with a community leader and public health advocate who has deep community ties and on-the-ground experience developing, advocating for, and implementing public health policies. These practitioners are particularly helpful in understanding mechanisms of policy action and important contextual factors as well as in advising on the recruitment of participants most knowledgeable about policy implementation.

#### 3.2.3 988 Lifeline

The project team is led by an experienced implementation scientist who focuses on policy and mental health. A statistician with substantive expertise in health policy impact analysis is integral to the team and brings deep expertise in methods related to causal inference and quasi-experimental policy impact evaluations. Given the project's focus on understanding variation across state 988 financing approaches–many of which are codified in statutes–a public health lawyer is a key member of the project team and integral to accurately specifying the different implementation financing approaches used by states. Heterogeneity in the effects of 988 financing strategies across demographic groups and equity considerations are core to the project, so a team member has expertise in racial and ethnic disparities in suicide and mental health crises. The team is also working with a public finance/accounting expert in a school of public administration to help measure and quantify financing. Finally, practice partners have been critical in helping the team stay abreast of the rapidly changing 988 financing and policy implementation environment.

### 3.3 Use mixed methods to identify, measure, and analyze relevant policy implementation determinants and processes

All three studies use mixed methods, though applied in different ways. Often, qualitative methods are used to understand implementation determinants, strategies, and variability across jurisdictions and implementing actors (30, 31). In all three projects discussed here, qualitative findings drive how policy implementation is measured and incorporated into quantitative policy evaluations and provide important context for quantitative findings.

#### 3.3.1 VAPOR

The VAPOR team is conducting interviews with staff in state agencies to understand how they are taking e-cigarette laws and translating them into action on the ground. The team's interview guides are based on Bullock's policy implementation framework, with significant focus on specific questions about determinants of implementation, for example, the clarity of policy language, the degree of vertical integration within state agencies, and the existence of communication and collaboration between state agencies and outside stakeholders (e.g., businesses). Along with the interviews, the team is asking each state to evaluate (a) the degree to which policies are implemented as written, and (b) whether they have adequate resources to implement the policies. The question is asked with respect to the initial implementation period and the current period, with responses taking values from 1 to 5. These numerical assessments will form the basis for the quantitative policy evaluation, allowing the team to move beyond “0/1” policy coding and determine whether policy impacts on study outcomes are different for states reporting “well-implemented” policies vs. “poorly implemented” policies. Interview data will provide further context on why policies do/do not show evidence of effectiveness, and will help the team decide how to approach the question of additive/multiplicative policies.

#### 3.3.2 CHECKOUT

The CHECKOUT investigators are conducting interviews, accompanied by brief quantitative surveys, with city staff, leaders, and community organizations involved in implementing the healthy checkout policy and with managers and owners of stores that are subject to the policy. The measures will characterize the implementation process and strategies, such as the overall implementation framework and timeline, the teams involved and their degree of coordination, training of inspectors, and communication of policy requirements to stores. Measures will also assess implementation outcomes, such as perceived acceptability of the policy, fidelity of enforcement, and costs of implementation, as well as other determinants of implementation, such as the complexity of policy requirements, presence of champions, prioritization of the policy, resources, and other barriers and facilitators (10, 18). A key implementation outcome–the extent to which stores comply with the policy–is being assessed quantitatively using repeated observations of products at store checkouts in Berkeley and comparison cities (32) and analyzed using difference-in-differences models. These observations of checkouts will be used to identify variability in compliance across stores and over time. Although at the time the evaluation was planned, there was only one city with a healthy checkout policy, another city, Perris, CA has since enacted a similar policy, and there are other jurisdictions also considering such policies. The quantitative measures used in this single-city study have the potential to be used in evaluations of subsequent healthy checkout policies, and the qualitative data will inform the expansion and refinement of quantitative measures.

#### 3.3.3 988 Lifeline

Because the 988 study is largely focused on one specific policy implementation financing strategy (i.e., state telecom fee legislation), the quantitative component uses difference-in-differences analyses to understand the impact of telecom user fee legislation on key implementation outcomes (i.e., measures of 988 implementation fidelity and reach–using call volume and routing data from Vibrant Emotional Health) and effectiveness outcomes (suicide death, using Centers for Disease Control and Prevention mortality data; suicide attempts, using self-report data from the National Survey on Drug Use and Health; and emergency department use for mental health crises and self-harm, using data from the Agency for Healthcare Research and Quality's State Emergency Department Databases). Moderation analyses will assess whether the state per capita amount of telecom fee revenue affects the relationship between user fee legislation and outcomes. Prior to the difference-in-difference analyses, surveys and interviews with “policy implementers” (e.g., 988 Lifeline call center leaders, state suicide prevention coordinators) will be conducted to unpack implementation processes and mechanisms of financing strategies. The information gained from these surveys and interviews may inform decisions in the difference-in-difference analyses (e.g., inform the selection and integration of new covariates) and will aid the interpretation of results. The surveys and interviews will draw from psychometrically validated instruments and assess stakeholders' perceptions of the financing determinants of 988 implementation and the acceptability and feasibility of state legislative financing strategies to improve implementation.

### 3.4 Build flexibility into project timelines to manage delays and challenges due to the real-world nature of policy

These projects' operationalization of implementation measurement also illustrates that policy implementation studies must wrestle with the real-world nature of policy implementation, which is constantly changing. This consideration is especially important when projects are evaluating policies as they are being implemented. The real-world dynamics of policy implementation work mean that investigators may need to consider backup plans in case data are delayed, unavailable, or change over time. Delays in policy implementation can run up against grant timelines, requiring no-cost extensions and even additional funding to sustain repeated measures longer than the initially anticipated need - though the three studies discussed herein are in early stages and have not yet faced these challenges.

#### 3.4.1 VAPOR

Policies affecting e-cigarettes differ across the US. VAPOR evaluations are happening over a five year grant period, and depending on the state, a policy might be recently enacted or amended, or long-standing. Repeated measures are thus critically important to fully understand implementation processes. One anticipated challenge that VAPOR has addressed is data acquisition. Some data on e-cigarette use is held by state departments of public health. Acquiring these data often requires obtaining state-specific data use agreements, which can be time-consuming to complete, file, and execute.

#### 3.4.2 CHECKOUT

Due to staffing shortages and strains on local governments caused by the COVID-19 pandemic, there has been a delay in some key aspects of the healthy checkout policy implementation, including the rollout of inspections and subsequent technical assistance to stores and fines. Such delays are not uncommon with municipal policy, and as such, policy implementation researchers need to be flexible and prepared to shift timelines for data collection and measurement (e.g., conducting interviews and surveys). Additionally, if government staff become too busy to participate in research, it may become necessary to rely on alternative data sources, such as public records and meeting minutes, to complement interview data. Potential delays in policy implementation also highlight the importance of collecting repeated measures of implementation outcomes. The researchers' multi-year assessments of products at checkout have the potential to detect increases in in-store compliance that may be expected immediately following policy communication, inspections, and fines (33).

#### 3.4.3 988 Lifeline

There has been more federal funding for 988 implementation than originally anticipated, and more states have been substantively supporting implementation through budget appropriations than projected. The research team has needed to modify their data collection approach to ensure that these funds are being adequately tracked and measured - including how much is being distributed to each state and how those dollars are being allocated. The 988 Lifeline has also expanded texting capacity, and thus, the team has had to revisit their initial analytic planning to make sure their variables and data appropriately capture text volume in addition to call volume. There is also a major upcoming change in how calls and texts are routed to local Lifeline centers and thus counted at the state level (34). Routing has been based on area code (high potential for measurement error or misclassification bias), and soon it will be based on geolocation (much lower potential for measurement error or misclassification bias). This is a great advancement for the real-world implementation and impact of the policy (i.e., callers will be routed based on their actual location, rather than their area code which does not necessarily reflect their current location) but poses a significant measurement challenge for the study because pre- and post-policy measures reflect different routing methods.

## 4 Discussion

It is critical that we incorporate policy implementation into quantitative evaluations of health policies. However, measuring policy implementation is a key gap in the literature. Indeed, while recent work has discussed how policy implementation is conceptualized in evaluations, less work has discussed how to operationalize and measure policy implementation, a prerequisite for including it in any analyses. The subfield of policy (or policy-focused) implementation science is well-poised to address this methodological gap in the literature (3, 16). Through group discussion and comparing the approaches and methods of three NIH-funded research projects, we identified four key design considerations for researchers to use to develop or identify measures of policy implementation for inclusion in quantitative analyses: (1) clearly specify the implementation logic of the policy under study, (2) develop an interdisciplinary team consisting of policy practitioners and researchers with expertise in quantitative methods, public policy and law, implementation science, and subject matter knowledge, (3) use mixed methods to identify, measure, and analyze relevant policy implementation determinants and processes, and (4) build flexibility into project timelines to manage delays and challenges due to the real-world nature of policy.

Our study reinforces the need for more work developing and validating transferrable measures of policy implementation determinants and outcomes (10). This represents a key area where implementation scientists with expertise in measure development and evaluation could greatly enhance policy-focused implementation science. Ideally, determinant and outcome measures would be transferrable across levels of policy (e.g., local, state, national), consistent within content areas, and include a focus on health equity (13–15, 35). Transferrable measurements will greatly enhance our ability to derive broadly generalizable knowledge from policy studies like those discussed here. Measures that are too study-specific will have limited generalizability (though potentially higher internal validity) and limit the ability of broader learning for the field of policy-focused implementation science. Including attention to health equity in measure development will help provide comprehensive understanding of how marginalized populations are impacted by policies (15). As measures are developed, improving the coordination and use of common measures through publicly available measure repositories is crucial to improving the efficiency, reproducibility, and learning potential of policy-focused implementation science research (15). Existing repository and field-building efforts can provide guidance for how to build and disseminate such repositories (36–38).

Prior systematic reviews have focused primarily on measures of policy implementation determinants and outcomes (10, 13, 14). Another area of research that needs to be expanded is understanding the process by which policy implementation occurs and how it unfolds over time. As one example, a number of studies have examined the process of implementing sugar-sweetened beverage taxes across multiple jurisdictions in the US. The collective impact has been to illustrate how the implementation of these tax policies varies by jurisdiction, including what implementation strategies were deployed across contexts (27, 39, 40). Another example investigated how three states chose to implement new substance use disorder care services under a Medicaid waiver policy and identified key implementation strategies deployed (31). As more work in the field studies policy implementation processes and identifies policy implementation strategies, ensuring that implementation strategies are clearly reported (24) and understanding the mechanisms by which implementation strategies affect implementation outcomes will be a critical next step, similar to work being undertaken in the broader field of implementation science (41).

Beyond reporting on successful implementation processes in the scientific literature, the timely and accessible sharing of this learning with policy- makers, writers, practitioners, implementers, and consumer protection organizations is key for disseminating best practices and informing future policy implementation efforts. Engaging policy practitioners as part of the research team is one avenue for timely dissemination. Also, in the process of recruiting policy implementers to participate in surveys and interviews, researchers can establish a preferred mechanism and format for the timely sharing of findings. This is crucial, as prior research has established that a “one-size-fits-all” approach to dissemination will likely not be successful (42–47). Another feedback loop through which the research can strengthen future policy implementations is by presenting to coalitions of public health policy practitioners. For instance, there is a national coalition of healthy retail policy practitioners that invites researchers to present findings that could inform their future policy work.

We urge researchers to be specific about the role that policy plays in their study, particularly when outlining the implementation logic that will drive project decisions. Policy can be conceptualized in many ways in implementation science, including considering policy as the “thing” of interest, policy as an implementation strategy to put an intervention into place, and policy as a “vessel” for interventions (3, 16, 21, 48, 49). Here, all three projects have a common goal of understanding strategies or processes by which policies were (or are being) put into place; the VAPOR and CHECKOUT studies conceptualized policies as the “thing” of interest, while the 988 Lifeline study conceptualized state policies as the implementation financing strategy deployed to support implementation of the federal 988 policy and increase the reach of Lifeline services. All are crucial lines of research that are needed to improve population health, but advancements in the field and collective learning will be impeded without conceptual clarity of the role of policy in individual studies.

A limitation of this work is that due to project constraints, we were limited to three case studies, all of which were in early stages during the development of this manuscript. Our intent was to provide illustrative considerations to measuring policy implementation but are not intended to be inclusive of all possible considerations for measurement, and we do not have evidence on the success of these considerations. However, we also note that our considerations overlap with related work in the policy and implementation science fields (6, 10, 13, 48, 50). For example, Crable et al*.* discuss the importance of clearly specifying a policy's form and function (48), similar to our suggested practice of clearly specifying the implementation logic of the policy under study using tools such as the IRLM. Second, our practice of considering mixed methods is consistent with considerations outlined in protocol papers from other teams involved in this area (51, 52), and reflections from authors in the field of public administration (53). Third, each of the studies is currently working to handle challenges in measurement because of the real-world nature of policy, consistent with findings from a report on other funded policy implementation studies (54).

## 5 Conclusions

Quantitative policy evaluations provide critical knowledge of how policies impact behavioral and health outcomes, building the evidence base for further adoption elsewhere. To appropriately evaluate policy impacts on health, we must adequately measure how the policy is implemented, rather than assuming a policy is implemented just because it is “on the books” (3). In turn, this can help researchers better understand the full picture of why policies do or do not affect health outcomes, and their impact on health disparities (3, 10, 15). Policy-focused implementation science research focuses on understanding just this, but the measurement of policy implementation is lacking. Here, we describe four design considerations for policy implementation measurement, particularly when researchers are seeking to include policy implementation quantitative evaluations of health policies. These considerations provide a foundation for the field to build on as attention to measuring policy implementation grows.

## Data Availability

The original contributions presented in the study are included in the article/Supplementary Material, further inquiries can be directed to the corresponding author.
