# Supplementary material for: Design considerations for developing measures of policy implementation in quantitative evaluations of public health policy
Source: Front Health Serv. 2024 Jul 15;4:1322702. doi: 10.3389/frhs.2024.1322702 (PMC11285065; doi:10.3389/frhs.2024.1322702)
Supplement: Supplementary file 1 [file Datasheet1.pdf]

Policy: State-level e-cigarette control policies: minimum age, taxes, indoor air, sales restrictions, licensure and other sales requirements

Study focus: Policy as the intervention **Policy level:** State policies

Determinants (CFIR)

Intervention Characteristics

Different for each of the various policies...
- Taxes: easy to adapt tax rate based on feasibility (+)
- Ability to level penalties (+)
- Issuance of retail licensure requirements
- Funding allocated for policy implementation (+)
- Complexity of policies and enforcement (-)
- Clarity of policy language (+)

Inner Setting

Main inner setting: state agencies
- Organizational structure; interagency relationships (+)
- Buy-in/push-back (+/-)
- Budget and staffing independent of the intervention (+)

Outer Setting

Main outer setting: state
- Buy-in/push-back from local governments, business, and consumers (+/-)
- State preemption laws/policy language (-)
- Support from NGOs (+)
- Public sentiment about smoking, tobacco use, e-cigarettes, and government regulation (+/-)
- Other tobacco-related legislation and events (e.g. for

Characteristics of Individuals

- Power/respect/trust in state tobacco control leadership (whose power/trust) (+)
- Skillsets of staff at all levels (+)

Process

Implementation Strategies

- Funding allocation when policy is unfunded or insufficiently funded by legislation

- Coordination among stakeholders within state government (e.g., within the health agency, between the health agency and licensing, revenue, and/or law enforcement agencies)

-Coordination across levels of government including with local or county governments as well as the federal government, including establishment of clear responsibilities

- Establish procedures and systems (including computer systems) for logistics and enforcement including agency staffing

- External communication, partnership, and education efforts with stakeholders (local governments, businesses, consumers). Efforts should be bidirectional. Those outside state government need to know what the policies are, what their roles are in enforcing, monitoring, or complying with the policies, and what the potential consequences are of not doing so. Communication should be bidirectional; external stakeholders should communicate with state government with insights about constituents, as well as anticipated barriers and facilitators.

Note: Strategies will be ascertained as the research progresses through the use of key informant interviews with state health agency personnel.

Mechanisms

Funding: Allows hiring of enforcement personnel (mediator or moderator)

Coordination: There are multiple entities that may be involved in licensure, operations, and enforcement. They will need to develop and maintain relationships and work together to achieve their implementation outcomes (mediator or moderator).

Internal communication and education efforts: clear workflows and effectively information sharing are conduits for effective policy execution (mediator).

External communication: external stakeholders will make the policy work when they know their roles and responsibilities and can share information with state agencies and other stakeholders (mediator).

Logistics: Enforcement will require adequate tracking systems in place, as well as mechanisms to apply and receive fines, revoke licenses as needed (mediator or moderator).

Penalties: Force retailers or end-users to change behavior (mediator).

Note: Generally factors in place independent of policy enactment (inner/outer setting) will be moderators, factors effected by the policy enactment will be mediators.

Outcomes

Implementation

- Tax revenues collected
- Enforcement actions taken
- Inspections/sting operations conducted
- Total budget for policy implementation and enforcement (legislated and non-legislated)

Service

Clinical/Patient

Primary: youth and adult e-cigarette use
Secondary: youth and adult use of combustible tobacco and cannabis

Adapted from the Implementation Research Logic Model (IRLM) from Smith, Rafferty & Li, 2020

Date:

Version:

Policy: Healthy checkout policy

Study focus: Policy as the intervention **Policy level:** Local policy (city of Berkeley, CA)

Determinants (CFIR)

Intervention Characteristics

•Simplicity (definitions of store, checkout, and eligible products) (+)
•Adequate period prior to enforcement (+)
•Ambiguity or omissions (multi-ingredient products, chips, standalone displays near checkout) (-)

Inner Setting

Main inner settings: CBO (funded to conduct initial retailer outreach), city government divisions (inspections, fines, communications, policy review), and retailers
•Funding for adequate staffing of CBO and city divisions (+)
•CBO and division understanding of role and responsibilities (+)
•Fit of inspections within city division workflow (+)
•Attitude toward legislation (retailers)

Outer Setting

Main outer setting: Berkeley
•Attitudes towards corporations and food system reform (Berkeley residents)
•Local conditions show health disparities, local history of food policy innovation and leadership, existing tax revenues from SSB tax, and university town

Characteristics of Individuals

•Individuals from CBO and city divisions conducting outreach
•Inspectors
•Retailers

Process

--

Implementation Strategies

Implementation strategies impact how the healthy checkout policy is implemented throughout Berkeley.

•Notification of affected retailers (identifying retailers and notifying each about the policy)

•Training and educating retailers (proactive training and outreach from CBO and city divisions, provision of technical assistance from city divisions)

•Regular inspections and levying of fines/warnings (training inspectors from the city division)

Mechanisms

These are the ways that implementation strategies impact outcomes:

•Retailer identification and notification increases the total number of retailers who comply with the policy and their degree of compliance

•Retailer training and education increases retailer knowledge, self-efficacy, and motivation to comply

•Regular inspections serve to increase retailer adherence to policy (to avoid fines/warnings)

•Funding from fines could serve to increase bandwidth for inspections

Outcomes

Implementation

Fidelity: Proportion of all products at checkout that are compliant across retailers and proportion of retailers that have a majority of their checkout products compliant

Service

Healthfulness of food environment

Clinical/Patient

Consumer purchases and dietary intake

Adapted from the Implementation Research Logic Model (IRLM) from Smith, Rafferty & Li, 2020

Date:

Version:

Policy: 988 Suicide & Crisis Lifeline, Transition to a New Three Digit Dialing Code

Study focus: Policy as the implementation strategy **Policy level:** Federal policy with state policy supporting implementation

Determinants (CFIR)

Intervention Characteristics

Easy to remember and of three digit dialing code (988) (+)

Builds on existing service with evidence base and reputation (+)

Inner Setting

Main inner setting: 988 call centers

- **988 Centers sufficiently staffed and funded (+)**
- 988 Center staff trained to deliver services in accordance with recommendations (+)

Outer Setting

Main outer setting: states

- **Availability of crisis services, system capacity (e.g., mobile crisis re**sponse teams, 24 hour crisis stabilization centers) (+)
- Public support for state telecom fee legislation (+)

Characteristics of Individuals

Wide range of crisis severity/acuity (callers)

Awareness of and trust in the Lifeline (potential callers)

Job satisfaction (staff)

Process

--

Implementation Strategies

State telecom fee legislation to create stable funding.

Fee legislation can take three main forms:

- **state telecom fee (yes/no)**
- **state 988 $ per capita per year**
- **federal 988 $ per capita per year**

Mechanisms

Fee legislation impacts outcomes through the following hypothesized mechanisms:

- $ for hiring, training, retaining staff in 988 Centers
- $ for quality 988 messaging/awareness campaigns
- $ for crisis services, system capacity (e.g., mobile crisis response teams, 24 hour crisis stabilization centers)
- Calls routed and fielded in-state of caller, supporting coordinated and quality care

Outcomes

Implementation

Fidelity: In-state answer rates

Reach: 988 call volume/contact rates

Service

Timely receipt receive of quality mental health crisis services for those in need

Clinical/Patient

- **Suicide attempts**
- **Suicide death**
- **ED visits for mental health crises and self-harm**

Adapted from the Implementation Research Logic Model (IRLM) from Smith, Rafferty & Li, 2020

Date:

Version:
